# Brain size affects the behavioural response to predators in female guppies (*Poecilia reticulata*)

**DOI:** 10.1098/rspb.2015.1132

**Published:** 2015-08-07

**Authors:** Wouter van der Bijl, Malin Thyselius, Alexander Kotrschal, Niclas Kolm

**Affiliations:** Department of Zoology/Ethology, Stockholm University, Svante Arrhenius väg 18B, Stockholm 10691, Sweden

**Keywords:** brain size, predator response, predator inspection, artificial selection, guppy, *Poecilia reticulata*

## Abstract

Large brains are thought to result from selection for cognitive benefits, but how enhanced cognition leads to increased fitness remains poorly understood. One explanation is that increased cognitive ability results in improved monitoring and assessment of predator threats. Here, we use male and female guppies (*Poecilia reticulata*), artificially selected for large and small brain size, to provide an experimental evaluation of this hypothesis. We examined their behavioural response as singletons, pairs or shoals of four towards a model predator. Large-brained females, but not males, spent less time performing predator inspections, an inherently risky behaviour. Video analysis revealed that large-brained females were further away from the model predator when in pairs but that they habituated quickly towards the model when in shoals of four. Males stayed further away from the predator model than females but again we found no brain size effect in males. We conclude that differences in brain size affect the female predator response. Large-brained females might be able to assess risk better or need less sensory information to reach an accurate conclusion. Our results provide experimental support for the general idea that predation pressure is likely to be important for the evolution of brain size in prey species.

## 1. Introduction

There is striking variation in both absolute and relative brain size at various taxonomic levels of vertebrates [1]. It is generally believed that this variation is generated through the balance of cognitive benefits of larger brains and costs of developing and maintaining a larger brain [2]. Exactly what the cognitive benefits of larger brains are, especially how these benefits relate to fitness, remain poorly understood.

The arms race between predator and prey can induce strong selection for a wide array of anti-predatory behaviours [3,4], and predator–prey interactions may also be important for brain size evolution. For instance, fossil records illustrate that ungulates that were in an arms race with carnivores evolved larger brains than those that were not [5]. Mammalian predators show consistent biases towards small-brained prey [6,7]. Additionally, bird species with relatively large brains can afford to delay their escape flight longer [8], and experience lower adult mortality in nature [9]. In an impressive analysis of 623 predator–prey pairs of fishes, Kondoh [10] found that: (i) the relative brain size of prey and predator were correlated, (ii) prey species had relatively larger brains than predator species, and (iii) prey–predator pairs could be identified with greater accuracy when brain size was taken into account. Together, these observations suggest that predation pressure can result in positive selection for relative brain size.

Fleeing from a predator may be expensive in terms of energy, increased visibility to other predators and missed opportunity costs, especially when threats are frequently encountered [11,12]. Therefore, prey are expected to try to predict the likelihood of an attack by a predator using monitoring and threat assessment and adjust their response appropriately. As this requires the gathering of information by the senses and then the processing of this information by the brain, it is likely that individuals with large brains, relative to their body size, would be better suited for this task [8].

Predator assessment is especially striking when prey actively approach the predator, as is found in a wide variety of animals. This has perhaps been best studied in small fishes such as minnows, sticklebacks and guppies [13]. These predator inspections usually consist of single fish or pairs swimming towards the predator in a tentative, saltatory manner [14]. During an inspection, fish are able to identify the predator, assess its attack motivation and anticipate strikes [15], and can use chemical cues to determine whether the predator's diet has included conspecifics [16]. Prey can then use this information to adjust their behaviour accordingly by increasing vigilance and reducing foraging [15], and to learn about the identity of novel predators [17]. At the same time, performing predator inspections is inherently risky behaviour. Some of this risk may be mitigated by avoiding the area around the mouth of the predator and approaching the flanks and rear instead, which is termed attack-cone avoidance [18]. Nonetheless, individuals with a high propensity to perform inspections are the first to get eaten [19], and the attack probability during inspection is a function of how close the inspector approaches [20–22]. Therefore, large brains would most likely provide a survival benefit under predation threat if they allow individuals to collect sufficient information based on fewer inspections or greater inspection distances.

Many animals living under the threat of predation form social groups to reduce the risk of mortality [23]. Guppies spend large portions of time shoaling closely with conspecifics [24] and the propensity to shoal covaries with local predator presence [25]. Even though predators preferentially attack larger guppy shoals owing to increased visibility [26], the *per capita* predation risk decreases with shoal size owing to increased vigilance [27], lower capture rates [26] and risk dilution. Additionally, the information gained by inspectors is, at least partially, transmitted to conspecifics who have not perceived the threat themselves [28]. Therefore, anti-predation behaviour is probably a function of the number of fishes, with smaller shoals being more cautious, and any brain size effects may not be apparent in all shoal sizes.

While the existing literature suggests a link between brain size and anti-predatory behaviour [5–10], it is correlational in nature and may be a result of other unidentified factors. For example, low levels of predation and extrinsic mortality may cause a shift in life-history strategy towards a longer lifespan [29], which in turn might favour larger brains [30]. In an attempt to exclude such latent variables, we examined the behavioural predator response of guppies (*Poecilia reticulata*) that have been artificially selected for relative brain size [2]. It has recently been shown in a large-scale survival experiment that large-brained females, but not males, from these selection lines survived better than small-brained individuals under predation threat in semi-natural conditions [31]. Based on the general assumption of greater cognitive abilities in large-brained individuals [2,32], we predict that large-brained individuals will require fewer inspections or be able to remain at larger distances from the predator. Additionally, we expect individuals in larger shoals to approach closer but perform fewer *per capita* inspections.

## 2. Material and methods

### (a) Animals and husbandry

The guppies used in the experiment were the result of four generations of replicated divergent selection for relative brain size (for details, see [2]). The starting populations for these lines were laboratory-reared descendants of Trinidadian guppies from high predation areas. Three times 75 pairs (75 pairs per replicate) were taken from these populations and allowed to breed. After the pairs had reproduced, they were sacrificed and their brains were weighed. Each pair was then assigned a score: the sum of their residuals of a regression of brain size on body size. The offspring from the highest and lowest scoring 25% of the pairs were then used to form new pairs, with whom selection continued. To avoid inbreeding, siblings were never matched.

After two generations of selection with this protocol (F_2_), the lines differed in relative brain size by on average 7.7% (males) and 9.0% (females; ranges: males 5.0–8.3%; females 8.0–9.3%) [2]. After three generations (F_3_), this average difference was 7.8% in males (range: 4.2–11.7%) and 10.3% in females (range: 8.5–11.9%). Offspring of those fish were then again paired (F_4_) according to their parent's relative brain size to produce F_5_. Measures of all brains of F_4_ are not obtainable owing to an ongoing longevity experiment. However, 36 F_4_ males not used for breeding were dissected and their relative brain weights differed on average by 13.8% (range: 10.5–19.8%; [32]). The fish used here are F_5_ and have therefore continuously been brain-weight-selected for four generations. As body size did not change in response to selection [2,33], both absolute and relative brain size were altered by the selection regime. The selection on brain size resulted in an improved performance in learning tasks for large-brained fish compared with small-brained fish [2,32].

After sexual maturation, the fish were kept in 50 l single-sex tanks, separated by replicate line and brain size. All fish used were fully matured and predator naive when the experiment began. Five days before the start of the experiment, the fish were moved into the room where trials were performed and placed in 7 l tanks of 14 individuals each. Animals were kept separated by brain size, replicate line and sex. They were fed with standard flake food at the end of each experimental day, kept under a 12 L : 12 D cycle and 27°C room temperature.

### (b) Treatment groups

We tested the two sexes, two brain sizes and three replicated selection lines in separate trials. As guppies often form small shoals in the field, and the predator response may vary by group size, we used singletons, pairs and shoals of four. The sexes, brain sizes and shoal sizes combined to a total of 12 treatment groups in a fully crossed design. Within each of those treatments, we tested 12 groups, for a total of 144 groups, using 336 fish (112 per replicate, see figure 2 and the electronic supplementary material, figure S1, for a breakdown of final sample sizes).

### (c) Trial set-up

The experiment started with transferring a shoal to an experimental tank (29 × 29 × 59 cm, 14 cm water level, aerated except during behavioural trials) in the afternoon and letting them acclimate overnight. The tanks contained white sand and the walls were covered with white PVC sheets to improve visibility of the fish on video. The next day, the fish went through a predator model trial and a novel object control trial, one in the morning and one in the afternoon. After testing, the fish were removed from the tanks and were not used again. Eight groups in eight separate tanks were tested on each day for a total of 18 days.

Before each trial, a camera (HD webcam C615, Logitech, Lausanne, Switzerland) was placed 1 m above the tank. After 20 min, either a predator model or a neutral novel object was gently placed in one end of the tank by hand. The behaviour of the fish was then recorded for 20 min at 30 frames per second. The predator model was one of two fishing lures (12 cm long; Lundgrens Fiskredskap, Sweden; electronic supplementary material, figure S5) custom-painted to resemble the pike cichlid (*Crenicichla alta*), a natural predator of the guppy. The model was attached to the bottom using fishing wire and a net covered with sand and floated upright. As a novel object control, we used a blue coffee mug that was placed on the bottom upside-down.

The different sexes, brain sizes, replicates, shoal sizes and predator model were randomly assigned to the testing days and time of day. The order in which predator and control were presented was also randomized. The experimenters were blind to which brain size and replicate the fish belonged to, with trials and tanks coded by running numbers. In two instances, the predator model and control cup were accidentally switched, rendering five trials (from different treatment groups) unusable.

### (d) Quantification of inspection behaviour

A single observer (M.T.) used the videos to manually score the start time, end time and group size of each predator inspection performed using JWatcher [34]. These data were then used to calculate the number of inspections, average inspection duration and the total time per fish that was spent inspecting. We scored an inspection when a guppy approached the model while keeping a visual fixation on the predator, swimming partly sideways with an arched body (‘avoidance drift’ *sensu* [35]). Often, individuals were seen using this sideways swimming to keep their distance to the model constant after they had approached the model. We had observed guppies performing this behaviour towards live pike cichlids behind glass in pilot experiments. The observer was blind to the replicate and brain size line that each trial belonged to.

### (e) Collection of positional data

We used computer vision software Ctrax [36] to track the exact position of the fish during the trials. All tracks were individually checked and corrected manually by a human observer (W.B.). Overall tracking performance was very reliable, although there were consistent problems in two areas of the tank. A glass ledge at one short edge of the tank provided poor visibility to fish underneath (10% of observations were located there), and fish were not distinguishable when directly swimming over or under the object placed in their tank (14% in control trials, 3% in predator trials). Observations in these areas were marked as missing data, except in the distance calculations (figure 3) where the observations at the model were included in the lowest bin.

As the tanks were filmed at a slight angle to avoid surface reflections, we used an image transform to project the coordinates to a rectangular plane [37]. As the items (predator models or cup) were placed in the tanks by hand and the desired position was judged by eye, there were slight deviations in item position between trials. To aid comparisons between trials of position relative to the object, and not relative to the tank, we used translation and rotation to overlay the objects.

### (f) Analysis, visualization and statistics

It was not possible for the human observer nor the computer vision software [36] to successfully keep track of individual identities over the course of a whole trial. Therefore, we consider all measurements a characteristic of the shoal and used shoal as the sampling unit. This procedure also enabled us to avoid pseudo-replication.

We fitted separate linear mixed-effects models (LMMs) to the three inspection variables and males and females. We used a power transformation for the time spent inspecting per fish and a log-transformation for number of inspections and mean inspection duration. Starting models included fixed effects for brain size, shoal size and their interaction as well as a random intercept and slope for brain size for each replicated selection line (*lme4* syntax for R: *y* ∼ brain size × shoal size + (brain size | replicate)). The interaction and main effect of shoal size were only retained if they considerably improved fit (Δ Akaike information Criterion (ΔAIC) > 2), brain size and the random term were always included. *p*-values for brain size and shoal size were computed using Satterthwaite's approximation for denominator degrees of freedom. The assumptions of normality and equality of variances were confirmed by visual inspection of the residuals.

We compared the occurrence of inspection (as a binary measure; ‘yes' or ‘no’) between control and predator model conditions with a generalized linear mixed model (GLMM) with logit link function, with a fixed effect for treatment (i.e. novel object versus predator model) and random effects for replicated selection line and shoal identification (id) (*lme4* syntax for R: *y* ∼ treatment + (1 | replicate) + (1 | shoal id)). We compared overall activity levels by calculating the average speed over the whole trial, which is directly related to the total distance travelled per fish.

We analysed the positional data by binning the observations in a grid, with cells being 0.5 × 0.5 cm large. For each trial, we calculated a density map, where the value for each grid cell was the fraction of all observations that occurred within that cell. This density map is a normalized representation of how often each part of the tank was visited by the fish. This means that, for each grid cell, each trial contributed a single value to the analysis. We then compared the independent data points within each grid cell to assess the effect of brain size. It is important to note, however, that the results between grid cells are not independent. This is because neighbouring cells are expected to be positively correlated as a fish can only reach a certain cell by travelling through others that are close-by. Moreover, the use of density as the statistic creates negative correlations between other cells as the sum of all cells is constrained to 1. These correlations somewhat alleviate the issue of multiple testing [38], but we still limit our interpretations to large scale patterns only.

Shoals typically did not visit every grid cell and occasionally fish would freeze in place for longer periods of time, up to several minutes. This means that the distribution of densities in a grid cell often contained many zeros and sometimes very high values, several orders of magnitude greater than the median. Fitting parametric models to the data within a cell would therefore be error prone [39]. To be confident that our conclusions were not disproportionally influenced by one or a few trials, we used non-parametric tests to assess significance. By using Wilcoxon signed-rank tests to compare densities to the median density, we could robustly visualize which areas of the tank were more or less frequently visited within each treatment. Additionally, we used Wilcoxon rank sum tests to statistically compare tank locations between the sexes and brain sizes. As we applied these tests to grids of observations, the outcomes are reported in graphical form (figure 2 and the electronic supplementary material, figure S1).

To still account for the replicates in the non-parametric analysis, we considered each set of replicates as an independent experiment of the same hypothesis and calculated three separate *p*-values for the effect of brain size. We then combined those using Stouffer's *z* method [40], a simple meta-analysis technique.

All analyses were performed using R statistical software v. 3.1.2 [41] with packages *lme4* v. 1.1–7 [42] and *lmerTest* v. 2.0–20 [43] used for mixed-effects modelling.

## 3. Results

The predator models were successful in evoking a predator response, as is evident in the large differences between predator and control trials (electronic supplementary material, figure S3; figure 2 versus electronic supplementary material, figure S1). Although we did observe some short inspections towards the neutral novel object, 83% of control trials did not have any inspections, versus 18% in predator trials (GLMM: *z* = 10.75, *p* < 0.001). Nevertheless, in some cases, fish seemed to resort to using the predator model as a refuge following an initial cautious response (e.g. figures 2*a* and 3*a*).

The overall rate of predator inspections (all predator trials: 1092 inspections in 48 h of observation = 23 inspections per hour) was similar to previous work with a live predator in shoals of four individuals ([21,22]: 166 inspections in 6 h = 28 inspections per hour, our data for shoals of 4: 504 in 15.7 h = 32 inspections per hour). We interpret this comparison as evidence for that our predator model and experimental design were successful.

**Figure 1.**
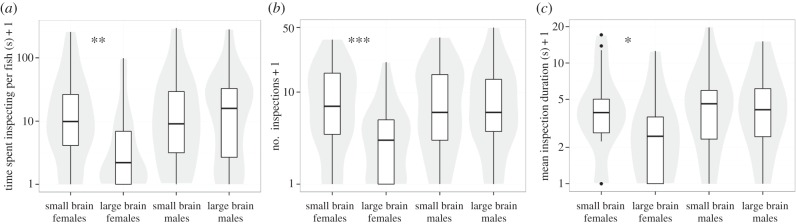
Parameters of inspection behaviour for the different treatments presented as boxplots, indicating the median and quartiles with whiskers reaching up to 1.5 times the interquartile range. The violin plot outlines illustrate kernel probability density, i.e. the width of the shaded area represents the proportion of the data located there. Significance is based on LMMs, see text and table 1 (*p < 0.05, **p < 0.01 and ***p < 0.001). (*a*) Total time spent inspecting the predator model per fish in the shoal. (*b*) Total number of inspections per shoal. (*c*) Average duration of those inspections. Note the use of log_10_ scales.

Females from small- and large-brained lines differed significantly in their propensity to perform predator inspections (figure 1 and table 1). Large-brained females spent much less time performing inspections than small-brained females (figure 1*a* and table 1), and this was true for all shoal sizes. This difference resulted from large-brained females performing both fewer (figure 1*b* and table 1) and shorter inspections (figure 1*c* and table 1). This effect of brain size in females was not the result of a difference in general activity, as the average speed was not affected by brain size (LMM: *F*_1,8.13_ = 0.16, *p* = 0.70). We observed no effect of brain size on male inspection behaviour (figure 1 and table 1). In females, larger shoals performed more inspections, but mean inspection duration and the time inspecting per fish were not affected by group size (table 1; electronic supplementary material, figure S4).

**Table 1.** Results from best models (LMM) on inspection behaviour, see the electronic supplementary material, table S1, for model selection results. (All models included random intercepts and slopes for brain size for each replicate. The two estimates for shoal size are for pairs and shoals of four, compared to the reference level of singletons. A power transform was applied to time spent inspecting per fish, and number of inspections and mean inspection duration were log-transformed prior to the analyses. Numbers in italics indicate significant values. *p < 0.05; **p < 0.01; ***p < 0.001.)

	fixed effect	estimate	s.e.	d.f. (approx.)	*F*	*p*
females						
time spent inspecting per fish	intercept	2.37	0.111			
	brain size	−0.47	0.157	1, 69	9.21	0.003**
number of inspections	intercept	1.49	0.216			
	brain size	−0.81	0.217	1, 67	13.85	<0.001***
	shoal size	0.30 / 0.82	0.26 / 0.27	2, 67	4.80	0.011*
mean inspection duration	intercept	1.32	0.125			
	brain size	−0.45	0.176	1, 69	6.59	0.012*
males						
time spent inspecting per fish	intercept	2.38	0.133			
	brain size	0.02	0.250	1, 2.04	0.007	0.940
number of inspections	intercept	0.09	0.084			
	brain size	0.01	0.152	1, 2.02	0.001	0.976
mean inspection duration	intercept	1.37	0.136			
	brain size	−0.02	0.263	1, 2.09	0.004	0.955

**Figure 2.**
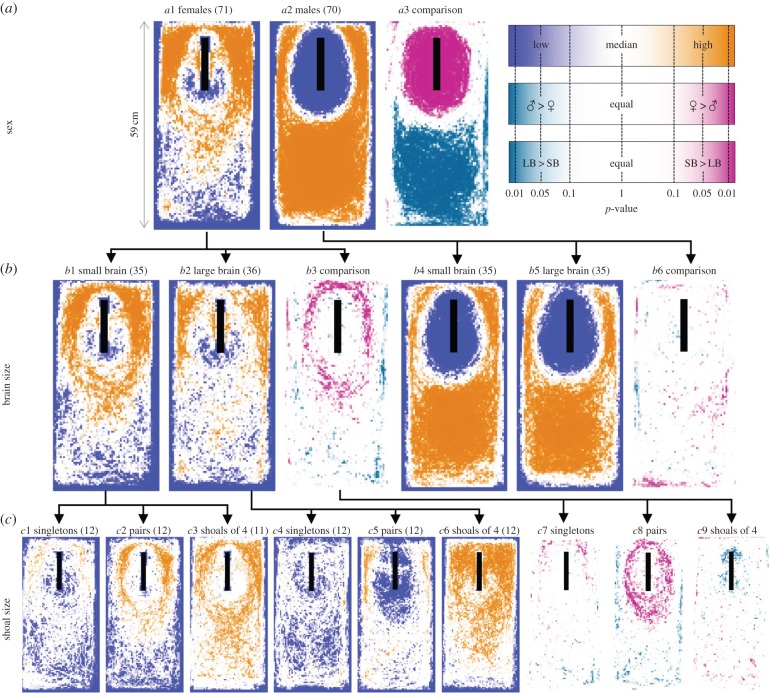
Overview of positional data during trials with a model predator, presented as statistical heat maps. First row (*a*) shows the overall result of each sex, second row (*b*) splits this up per brain size, and the third row (*c*) divides the brain size results for females up into the different shoal sizes. Each cell (pixel) in the heat maps shows the *p*-value of a non-parametric test. In the orange and blue maps, we tested against the median density to visualize the areas of the tanks that were visited more or less than expected. The pink and green maps show the statistical comparison between groups; i.e. males and females or small- and large-brained individuals. Black rectangles indicate predator position. Numbers between parentheses denote sample sizes. Note the nonlinear axis in the legend.

All groups showed a reduced presence in the closer proximities around the predator model (figure 2). Typically, we observed that fish spent most of their time in an elliptical ring around the predator model at approximately 10 cm distance. While this ring was strongly present in the first 5 min, it would typically become less pronounced later in the trial (electronic supplementary material, figure S2). Males were much more cautious and rarely ever came within that ellipse (figure 2*a*2), while females did occasionally approach at closer distances (figure 2*a*1). This leads to a strong difference in tank location between the sexes, with the entire region around the predator model being visited more frequently by females (figure 2*a*3). Females especially got close to the rear and sides of the predator model, but rarely to the head (figure 2*a*1), indicating they employed attack-cone avoidance [18].

**Figure 3.**
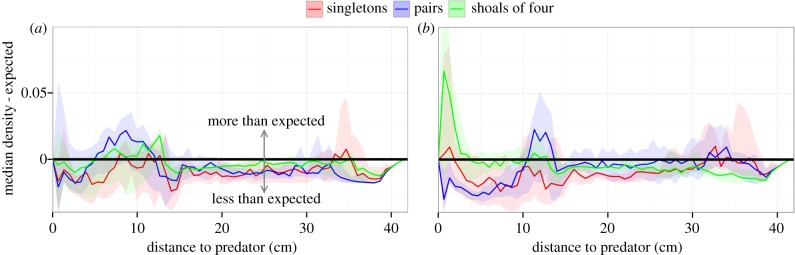
Density distributions of the distance to the predator for (*a*) small- and (*b*) large-brained females, split by shoal size (colours). As each trial has an individual density distribution, lines represent the median of those distributions for each group with the shaded areas being bootstrapped 95% CIs. As the expected distance distribution is determined by the shape of the tank, relative density distributions are shown, where the expected distribution (when tank locations would be drawn randomly from a two-dimensional uniform distribution) has been subtracted.

Small-brained females had high presence in a full ring around the predator model (figure 2*b*1), whereas large-brained females tended to spend more time at the flanks of the predator, close to the edge of the tank (figure 2*b*2). We found a significant difference in a ring-pattern relatively close to the predator, where small-brained females resided more often (figure 2*b*3). When the brain size comparison was split up by shoal size, it was apparent that this difference was mostly driven by the large effect of brain size in groups of two. In those pairs, large-brained females stayed much further away from the predator than small-brained females (figure 2*c*2,*c*5), and the entire area around the predator had higher presence for small-brained female pairs (figure 2*c*8). The same was true for singletons, but the statistical comparison was hindered by the low amount of data (figure 2*c*7). In shoals of four, differences were small, but large-brained females came close to the predator model more often, especially to the rear of it (figure 2*c*9). When the data were split into four 5 min periods, it was apparent that the large-brained females did show a more cautious response to the predator model, characterized by the high presence ring around the predator model, at the start of the trial. However, this pattern disappeared at later times (electronic supplementary material, figure S2, bottom). Shoal size affected the distance that was kept from the predator more in large-brained females than in small-brained females (figure 3*a*,*b*).

When females were confronted with the coffee mug as a novel object control, individuals were found most often in close proximity to the mug or near the walls (electronic supplementary material, figure S1a1). There were no detectable brain size differences in control trials (electronic supplementary material, figure S1b3). In males, we found no evidence of an effect of brain size on position in the tank in either predator model (figure 2*b*6) or neutral novel object (electronic supplementary material, figure S1b6) situations.

## 4. Discussion

Large-brained females responded very differently than small-brained females towards the predator model, both in terms of inspection rates and tank location, whereas males showed no differences between large- and small-brained lines. Overall, males acted more cautiously than females, which could be owing to a difference in ecological relevance of the set-up for the sexes. We further found inspection rates of individuals to be independent of shoal size. We discuss below how the brain size effect in females may relate to differences in personality and propose that mainly improvements in cognitive traits in large-brained females, such as perception and decision-making, underlie these results. We further develop the argument that large-brained females showed a high level of adaptive behavioural flexibility by adjusting their tank location to the shoal size. We finally discuss how our results support the hypothesis that predator response is an important factor in the evolution of vertebrate brain size and the general implications of our findings.

Strong differences can be seen, both in inspection behaviour and tank location, in trials where guppies were confronted with a model predator versus trials where they were shown a neutral novel object. This confirms that the models were perceived as a threat.

Males, on average, kept a larger distance from the predator model than females, although females may have mitigated some of the risk by avoiding the cone of attack. This is consistent with field observations [35], where females also approached closer and avoided the mouth region more than males. This behaviour might be explained by the fact that, compared with females, male guppies face higher mortality risk owing to their brightly coloured sexual ornamentation [44]. While males kept a larger distance to the control object as well, an earlier study of these guppies found no sex difference in how much time individuals spent in the centre of an open field [33]. We note that the larger females generally have to balance predator awareness with foraging behaviour to increase fecundity, while investing time and energy in sexual behaviour is more important for males [45]. As we used single-sex shoals, our set-up did not force males into trading off vigilance with sexual investment while females may still have traded vigilance against time spent searching for food. If vigilance was cheap for males, this could have resulted in males being more vigilant than females. Additionally, if all males were vigilant, an effect of brain size would be hard to detect. Alternatively, the sex difference in location could have originated in males perceiving the predator models as lower risk compared to the females, resulting in little need to make approaches. However, we deem this unlikely as males and females showed similar inspection rates. If anything, the opposite might be the case as males showed a larger difference in location between the control trials and predator model trials than females.

As the information gained by inspections can be shared among the shoal [28], we expected that fish in larger shoals would decrease the time spent inspecting. Our results show, however, that the total number of inspections of the shoal increased as an effect of increased shoal size and individual fish did not significantly alter their inspection rate. While some information can be obtained from conspecifics, the information that an individual gains from itself inspecting is likely to be more detailed or reliable [28] and may make inspecting worthwhile, especially if inspecting in larger groups is already less costly because of dilution of risk [20,26]. This is in agreement with the observation that when minnows only receive social information of predation threat, they reduce foraging, but they do not increase skittering, where fish rapidly disperse, which is thought to be expensive [28].

Artificial selection on brain size affected the predator response of female guppies, but not of males. The propensity to inspect the predator was strongly reduced in large-brained females and this was accompanied by an overall difference in their location in the tank. In contrast to the inspection rates, however, the effect of brain size on tank location in females was not consistent between different shoal sizes. While the large-brained females seemed cautious when alone or in pairs, which may be indicated by them keeping their distance, this behaviour radically changed when in shoals of four. There, they only briefly maintained a safe distance before losing caution and starting to use the model as refuge instead. Small-brained females, on the other hand, remained at similar distances regardless of shoal size. This suggests that the heterogeneity in the effect of brain size on tank location in females was at least partly owing to large-brained females exhibiting a greater capacity of plasticity in their response. As adjusting behaviour to a situational cue is a fundamental element of cognition [46,47], we speculate that this flexibility in large-brained guppies generalizes to other domains. As it would be beneficial to vary anti-predation behaviour with predation risk and the *per capita* mortality risk decreases with shoal size [26], the behavioural flexibility shown by large-brained females is likely to be adaptive.

Could the effect of brain size in females reflect a general difference in personality? We may expect general personality traits that are important in challenging situations, such as ‘boldness', to be of predictive value to the predator response. Previous work in these selection lines has shown large-brained females to be bolder, when quantified as time spent in the centre of an open field [33]. If boldness was an important factor in the predator trials, we would therefore have expected large-brained females to show more inspections and approach closer to the predator. Instead, the large-brained females inspected less and selectively approached closer in shoals of four, but not as singletons or pairs. Moreover, we did not observe any differences between individuals of different brain size in the novel object control trials, also often used to assess boldness [48]. Therefore, it seems that, in guppies at least, the open field and novel object tests do not predict the predator response. We caution against the extrapolation of boldness measured in general personality tests towards predation situations, in absence of extensive validation in the species of interest (in line with [49]). At the same time, while it remains unclear how our results would relate to any particular personality axis, we expect certain aspects of personality to be affected by brain size as a change in cognitive ability would affect a suite of behaviours across contexts and time [33].

As in any artificial selection experiment, the differences that we find could be the result of co-selected traits instead of the trait of interest. Specifically, earlier work has shown that large-brained fish have a lower cortisol response to confinement stress [33]. This hormonal difference may contribute to a difference in behaviour in a stressful situation such as a predator encounter, although we would then expect results in the opposite direction from what we found here, assuming higher stress levels invoke a stronger avoidance. Other traits, such as metabolic rate, may also be important and remain to be investigated.

We hypothesize that the brain size effect in females was mainly driven by differences in cognitive traits. Components of cognition (*sensu* [50]) that may aid in the predator response are perception, working memory, attention and decision-making. It would be challenging to convincingly demonstrate which of these are directly affected by brain size and subsequently affect the studied behaviour, but it is conceivable that a larger optic lobe would facilitate visual perception, and a larger telencephalon could increase the accuracy or speed of a decision. It is unclear whether large-brained females may pay the price of their reduced inspections by not acquiring benefits of such behaviour, such as learning the identity of novel predators [17]. These potential benefits could still be obtained if their large brains allow for a better learning performance or the collection of equal amounts of information from larger distances. The experiments here examined only the first encounter with a predator of naive fish, and therefore we did not test for any effects facilitated by learning and long-term memory. We expect that the effect of brain size would increase with further encounters, as there are numerous opportunities for learning throughout the predation sequence [51], and large-brained individuals have been shown to outperform small-brained individuals in learning tasks that placed demands on memory [2,32].

Since predator inspection, a well-studied and stereotypical behaviour, puts the animal at risk and is associated with increased mortality [19,20], the reduction of inspection rate that we observed in large-brained females is predicted to lead to a survival benefit of individuals with large brains in the presence of predators. Indeed, the results from a large-scale survival experiment using the same selection lines show that females, but not males, survived better under predation threat in semi-natural conditions [31]. We suggest that differences in the behavioural predator response form a plausible mechanism behind this effect, especially as the brain size effects on distance and inspection rate are also restricted to females.

As the experiments here were performed with predator-naive fish, we view the predator model as a novel environmental challenge. Successfully responding to such challenges is at the core of the cognitive buffer hypothesis, as is the ability to flexibly change behaviour [52]. The brain size effects described here and the plasticity of the response of large-brained females in regards to shoal size support that the general adaptive value of large brains extends to predator–prey interactions. Therefore, we expect that future comparative analysis between species, as well as population comparisons within species, may show that large brains are associated with higher predation pressure, and possibly certain strategies used by the prey or the predator. The strong differences in predation pressure between guppy populations within streams in Trinidad, where predators are present below waterfalls but have not extensively colonized the pools upstream [25], could be used to test this prediction. Interestingly, a tell-tale sign of a population under predation threat is the formation of social groups, and group size covaries with relative brain size in primates [53] and ungulates [54], but not in carnivores [55]. The link between group size and brain size, which is typically attributed to the high cognitive demands of social interactions [56], may thus be a side-effect from adaptation in response to predation.

In conclusion, the artificial selection on relative brain size led to distinct alterations in the predator response of female guppies, while we found no effects in males. Future work on the relationship between brain size, social processes and group decisions may help us identify the mechanisms underlying the dependency of the location effect on shoal size. While it is unlikely that any single ecological variable can explain the wide interspecific variation in brain size, responding to the threat of predation may result in an important positive selection pressure for prey species. Hence, our results highlight predator response as a potentially important factor for the formation and maintenance of variation in brain size.

## Data accessibility

Data is available on Dryad, http://dx.doi.org/10.5061/dyrad.t741c.
